# Supplementary material for: Identification of N-Acetyl-S-(3-Cyano-2-(Methylsulfanyl)Propyl-Cysteine as a Major Human Urine Metabolite from the Epithionitrile 1-Cyano-2,3-Epithiopropane, the Main Glucosinolate Hydrolysis Product from Cabbage
Source: Nutrients. 2019 Apr 23;11(4):908. doi: 10.3390/nu11040908 (PMC6521188; doi:10.3390/nu11040908)

Supplemental Information 1: ^{1}H- and ^{13}C-NMR data of *N*-Acetyl-*S*-(3-cyano-2-(methylsulfanyl)propyl-cysteine

The substance is an approximate 1:1 mixture of 2 diastereoisomers:
N-Acetyl-*S*-(3-cyano-2*R*-(methylsulfanyl)propyl-cysteine
N-Acetyl-*S*-(3-cyano-2*S*-(methylsulfanyl)propyl-cysteine

^{1}H-NMR spectrum
^{1}H NMR (400 MHz, $CDCl_3$) δ ppm: 6.75 + 6.73 (2 br s, 1 H), 6.42 (br s), 4.86 - 4.79 (m, 1 H), 3.21 - 2.95 (m, 4 H), 2.75 - 2.92 (m, 3 H), 2.21 +2.20 (2 s, 3 H), 2.11 (s, 3 H)

^{13}C-NMR spectrum
^{13}C NMR (101 MHz, $CDCl_3$) δ ppm: 172.5 (1C), 171.8 + 171.7 (1C), 117.4 + 117.3 (1C), 52.4 + 52.3 (1C), 42.6 + 42.4 (1C), 36.8 + 36.8 (1C), 34.6 + 34.4 (1C), 22.9 (1C), 22.7 (1C), 14.3 + 14.2 (1C)

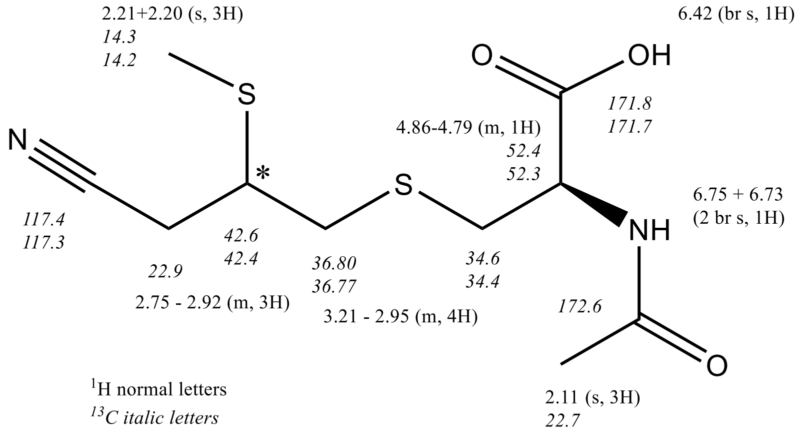

^{1}H normal letters
^{13}C italic letters

1H-NMR spectrum

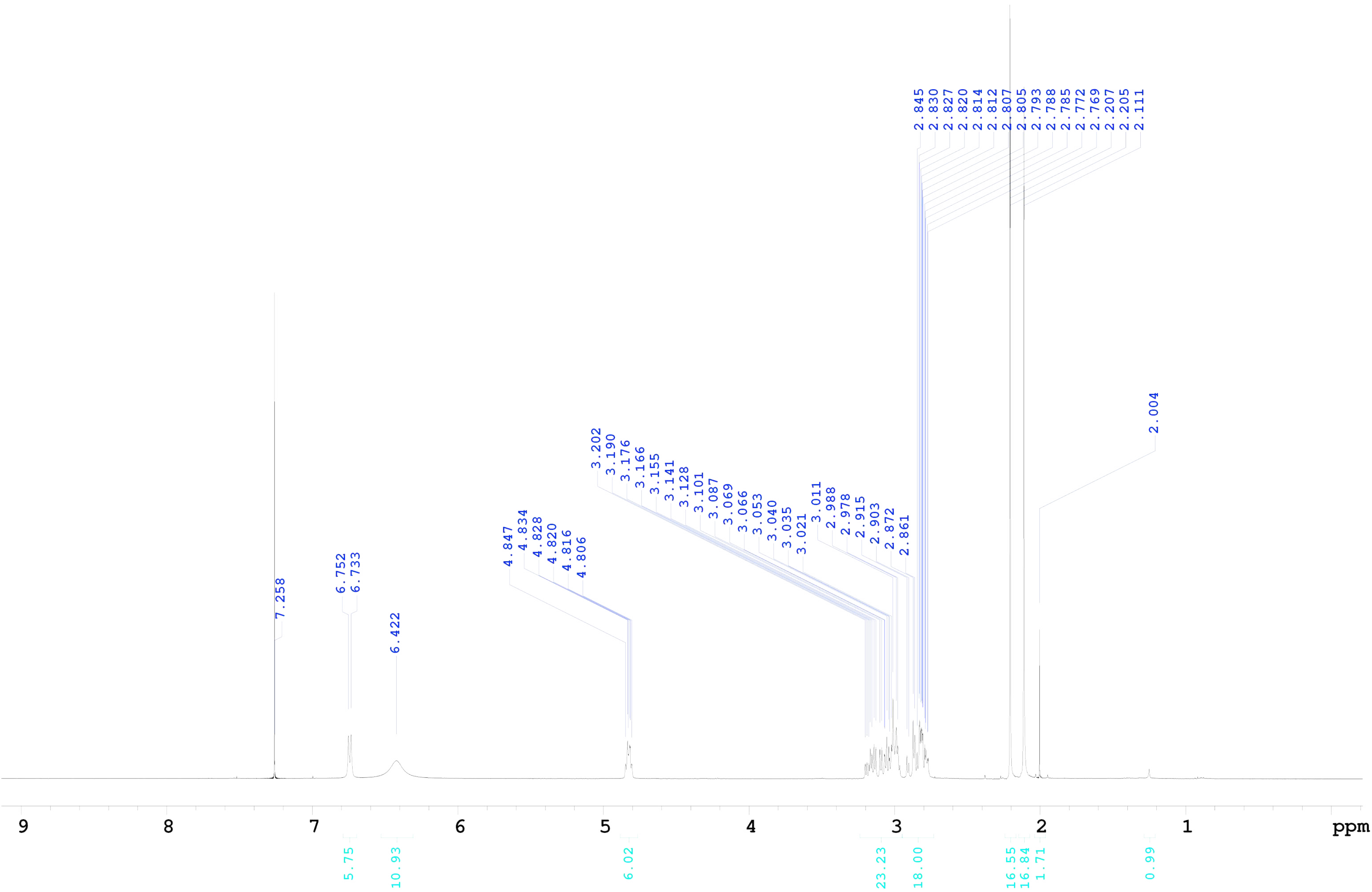

13C-NMR spectrum

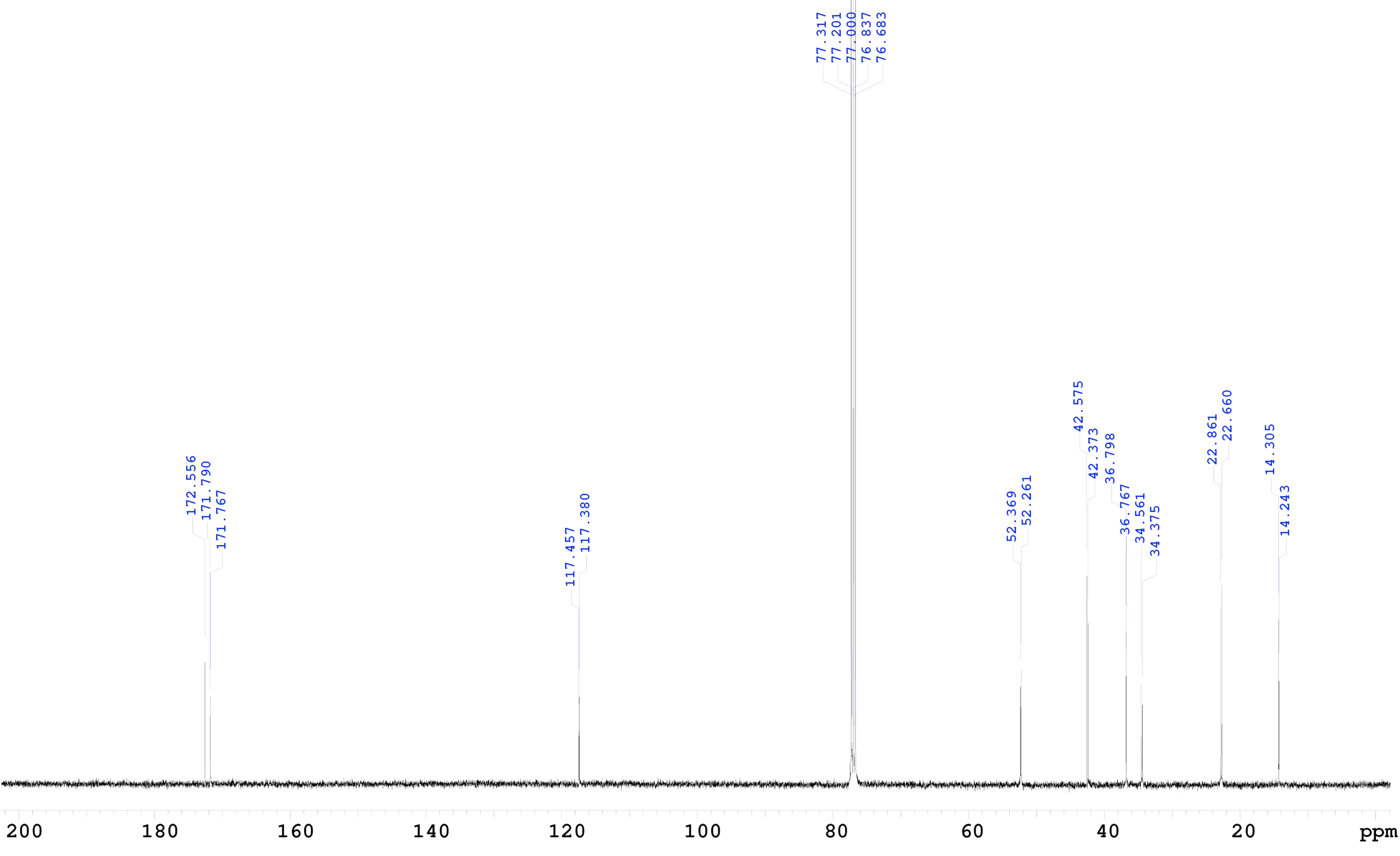

Supplement: Supplementary file 1 [file nutrients-11-00908-s001.zip › Supplemental Information 1_NMRmerged.pdf]
